# Diagnostic ability and sectoral structure–function relationship of circumpapillary and macular superficial vessel density in early glaucomatous eyes

**DOI:** 10.1038/s41598-022-10033-1

**Published:** 2022-04-09

**Authors:** Kaho Akiyama, Hitomi Saito, Shiroaki Shirato, Aiko Iwase, Koichiro Sugimoto, Takashi Fujishiro, Hiroshi Murata, Rei Sakata, Megumi Honjo, Makoto Aihara

**Affiliations:** 1 Department of Ophthalmology, University of Tokyo, 7-3-1 Hongo, Bunkyo-ku, Tokyo, 113-8655 Japan; 2 Yotsuya Shirato Eye Clinic, 1-1-2 Yotsuya, Shinjuku-ku, Tokyo, Japan; 3 Tajimi Iwase Eye Clinic, 3-101-1 Honmachi, Tajimi-shi, Gifu, Japan

**Keywords:** Glaucoma, Optic nerve diseases

## Abstract

This study aimed to evaluate the diagnostic ability and sectoral structure function relationship of circumpapillary vessel density (cpVD) and macular vessel density (mVD) with optical coherence tomography angiography (OCTA) in early glaucomatous (EG) eyes. 224 EG eyes of 167 patients (mean deviation (MD) > − 6 dB) and 70 normal eyes of 70 subjects were enrolled in this retrospective cross-sectional study. All patients underwent OCT and OCTA scanning. Diagnostic abilities were evaluated with area under receiver operating characteristic curves (AUROC). Structure function relationships of superior, inferior and Garway-Heath sectoral values with its corresponding visual field (VF) sensitivity were determined using linear mixed models. AUROCs were 0.798, 0.621, 0.876 and 0.835 for cpVD, mVD, circumpapillary retinal nerve fiber layer thickness (cpRNFLT) and ganglion cell-inner plexiform layer, respectively. AUROC of cpVD was significantly lower than cpRNFLT (*P* = 0.010) and higher than mVD (*P* < 0.001). All Garway-Heath sectors of cpVD significantly correlated with its corresponding VF sensitivity except for the nasal sector. MVD also showed significant structure function relationship and the correlations were stronger in the perifoveal region (6 mm annulus) than in the parafoveal region (3 mm annulus). CpVD demonstrated moderate diagnostic ability and both cpVD and mVD demonstrated significant association with VF sensitivity in EG eyes.

## Introduction

Glaucoma is a chronic progressive optic neuropathy characterized by visual field damage (VFD), and is the second leading cause of blindness worldwide^1^. As glaucomatous damage is irreversible, early diagnosis and treatment are essential in slowing progression of VFD. Although fundoscopic optic disc observation and visual field (VF) testing remain the standard criterion for glaucoma diagnosis and assessment^2,3^, novel imaging technologies play an increased role in detecting glaucomatous damage especially in early glaucomatous eyes in which clinical diagnosis is most difficult.

Previous studies reported an association between vascular compromise and glaucomatous VFD using various techniques such as laser speckle flowgraphy, laser doppler flowmetry and fluorescein or indocyanine angiography^4–9^. Optical coherence tomography angiography (OCTA) is one of the most recent optical coherence tomography (OCT) technologies which enables non-invasive imaging to quantify layer specific ocular microcirculation of the retina and choroid. With the advancement of technology, the newest OCT models have achieved more rapid OCTA imaging and acquisition of higher quality images with a wider field of view allowing better assessment of ocular circulatory changes.

The diagnostic ability of circumpapillary vessel density (cpVD) and macular vessel density (mVD) in glaucoma patients of different glaucoma severity have been reported using several OCTA instruments^10–19^. In addition, OCTA has been shown to be able to detect focal glaucomatous changes^20^, which is key to diagnosing early glaucoma. However, reports on the diagnostic ability of OCTA in early glaucomatous eyes, in which the help of OCT is most crucial, were mainly conducted on very small numbers of patients and the results were varying^11–17^. Previously reported diagnostic ability of OCTA parameters ranged from moderate to high (AUROC: 0.726 to 0.965) for cpVD and low to high (AUROC: 0.562 to 0.92) for mVD. Furthermore, which OCT parameter best discriminates early glaucomatous eyes from normal eyes is still inconclusive, leaving the role of OCTA in the earlier stages of glaucoma controversial^11–14^. The purpose of this study is to evaluate the diagnostic ability and to explore focal sectoral structure function relationships of cpVD and mVD measured by OCTA in early glaucomatous eyes in a large cohort of eyes using the newest model OCTA.

## Results

In total, OCTA scans were obtained from 294 eyes of 214 glaucoma subjects and 147 eyes of 76 normal subjects. After excluding eyes which did not meet the inclusion criteria, 224 eyes of 167 glaucoma subjects and 70 eyes of 70 normal subjects were included in this study. Table 1 presents background information of the study subjects. There was no significant difference in age, sex and axial length between the normal and glaucomatous eyes. Average mean deviation (MD) of the glaucomatous eyes (including 41 preperimetric glaucoma (PPG) eyes) was − 2.01 ± 1.94 dB.

**Table 1:** Demographic and clinical characteristics of glaucomatous eyes and normal eyes.

	Glaucoma (n = 224)	Normal (n = 70)	*P* value
Age (years old)	55.7 ± 12.5	53.6 ± 15.1	0.283*
Sex (male/female)	108/116	35/35	0.622^†^
Axial length (mm)	25.1 ± 1.4	24.8 ± 1.6	0.088*
Visual acuity (log MAR)	− 0.071 ± 0.027	− 0.074 ± 0.022	0.355*
Intraocular pressure (mmHg)	13.5 ± 2.5	14.7 ± 3.0	0.001*
Mean deviation (dB)	− 2.01 ± 1.94	− 0.06 ± 1.56	< 0.001*
Pattern standard deviation (dB)	4.70 ± 3.12	1.70 ± 0.41	< 0.001*

log MAR: log minimum angle of resolution.

Significant values are shown in bold.

*Un-paired t-test.

^†^Chi-square test.

Intra-visit reproducibilities of the angiography scans were 0.969 and 0.750 for global cpVD and mVD, respectively. Global cpVD, circumpapillary retinal nerve fiber layer thickness (cpRNFLT), mVD and ganglion cell-inner plexiform layer (GCIPL) were significantly lower in glaucomatous eyes (Table 2). Superior, inferior, and all Garway-Heath sectors except for the superior nasal sector of cpVD and nasal sector of cpRNFLT were significantly lower in glaucomatous eyes (Table 2). Perifoveal inferior mVD was significantly lower in glaucomatous eyes while there were no statistical differences in the parafoveal mVD sectors (Table 2). Average, superior and inferior GCIPL were all significantly thinner in glaucomatous eyes (Table 2).

**Table 2:** CpVD, cpRNFLT, mVD and GCIPL measurements of glaucomatous eyes and normal eyes.

		Glaucoma (n = 167)	Normal (n = 70)	*P* value*
cpVD (%)	Average	42.8 ± 2.1	44.9 ± 1.6	** < 0.001**
	Superior	41.6 ± 3.5	43.1 ± 2.3	**0.016**
	Inferior	40.8 ± 3.4	44.7 ± 2.1	** < 0.001**
	Inferior nasal	41.5 ± 3.6	43.3 ± 3.0	**0.003**
	Inferior temporal	40.1 ± 6.0	45.7 ± 2.2	** < 0.001**
	Superior nasal	41.6 ± 4.0	41.9 ± 3.2	0.894
	Superior temporal	42.0 ± 5.1	44.7 ± 2.8	**0.001**
	Nasal	42.3 ± 2.2	43.2 ± 2.1	**0.013**
	Temporal	46.0 ± 3.0	48.2 ± 2.1	** < 0.001**
cpRNFLT (µm)	Average	75.7 ± 8.6	90.0 ± 8.7	** < 0.001**
	Superior	89.8 ± 17.2	106.5 ± 16.3	** < 0.001**
	Inferior	83.9 ± 17.6	112.7 ± 15.9	** < 0.001**
	Inferior nasal	80.0 ± 16.4	94.4 ± 20.2	** < 0.001**
	Inferior temporal	90.4 ± 28.6	136.4 ± 19.5	** < 0.001**
	Superior nasal	84.6 ± 18.7	94.1 ± 20.8	**0.007**
	Superior temporal	96.3 ± 25.5	123.1 ± 20.4	** < 0.001**
	Nasal	68.7 ± 8.9	69.1 ± 9.5	0.796
	Temporal	63.6 ± 12.1	76.1 ± 13.8	** < 0.001**
mVD (%)	Average	44.4 ± 4.4	46.0 ± 2.4	**0.040**
	Perifoveal superior	46.2 ± 4.3	47.3 ± 2.7	0.218
	Perifoveal inferior	43.1 ± 6.8	47.6 ± 3.2	** < 0.001**
	Parafoveal superior	46.2 ± 3.6	45.7 ± 3.6	0.122
	Parafoveal inferior	45.1 ± 5.3	46.0 ± 3.0	0.481
GCIPL (µm)	Average	68.7 ± 8.0	78.6 ± 6.1	** < 0.001**
	Superior	71.3 ± 9.1	79.1 ± 6.2	** < 0.001**
	Inferior	66.2 ± 9.5	78.2 ± 6.5	** < 0.001**

cpVD: circumpapillary vessel density; cpRNFLT: circumpapillary retinal nerve fiber layer thickness; mVD: macular vessel density; GCIPL: ganglion cell-inner plexiform layer; ANCOVA: analysis of covariance.

Significant values are shown in bold.

*ANCOVA adjusted for age and axial length.

**Figure 1:**
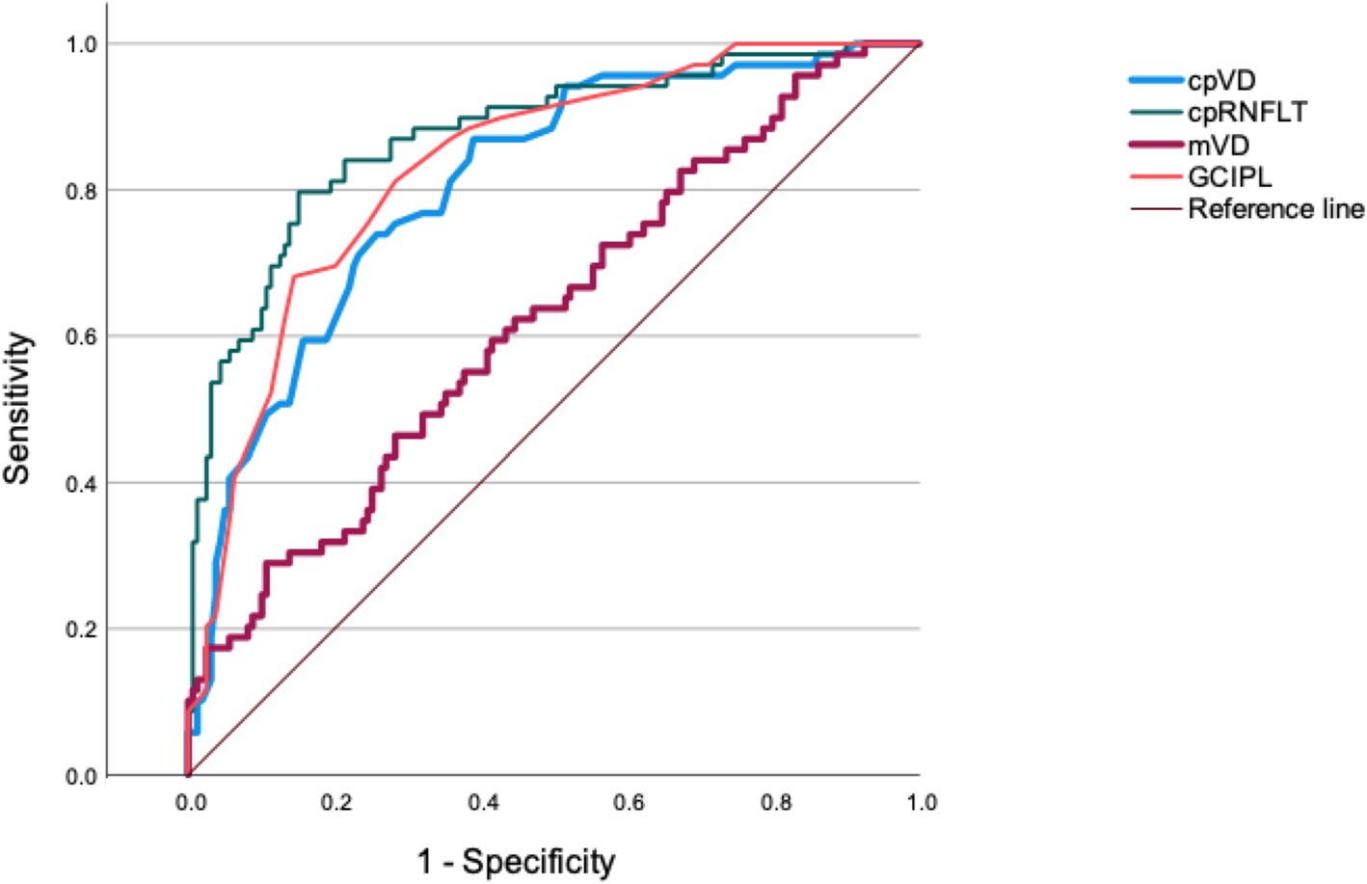
Receiver operating characteristic (ROC) curves of cpVD, cpRNFLT, mVD and GCIPL for discriminating glaucomatous eyes from healthy eyes. Area under the ROC was 0.798 (95% confidence interval (CI) 0.737 to 0.858) for cpVD, 0.876 (95% CI 0.825 to 0.928) for cpRNFLT, 0.621 (95% CI 0.542 to 0.701) for mVD, 0.835 (95% CI 0.781 to 0.890) for GCIPL. cpVD: circumpapillary vessel density; cpRNFLT: circumpapillary retinal nerve fiber layer thickness; mVD: macular vessel density; GCIPL: ganglion cell-inner plexiform layer.

ROCs of the 4 parameters are presented in Fig. 1. AUROC was highest with cpRNFLT (0.876; 95% confidence interval (CI) 0.825 to 0.928), followed by GCIPL (0.835; 95% CI 0.781 to 0.890), cpVD (0.798; 95% CI 0.737 to 0.858) and mVD (0.621; 95% CI 0.542 to 0.701). AUROC of cpVD was significantly higher than that of mVD (*P* < 0.001), but lower than that of cpRNFLT (*P* = 0.010). There was no statistical difference between AUROC of cpVD and GCIPL (*P* = 0.257).

**Figure 2:**
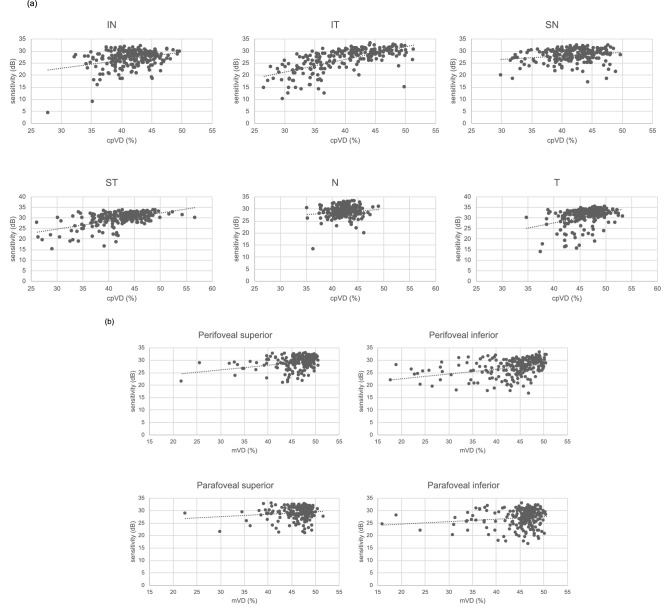
(**a**) Scatter plots of sectoral cpVD (%) and corresponding VF sensitivity (dB). (**b**) Scatter plots of sectoral mVD (%) and corresponding VF sensitivity (dB). cpVD: circumpapillary vessel density; mVD: macular vessel density; IN: inferior nasal; IT: inferior temporal; SN: superior nasal; ST: superior temporal; N: nasal; T: temporal.

Table 3 presents the structure function relationships of sectoral cpVD, cpRNFLT, mVD and GCIPL with its corresponding VF sensitivity in the glaucomatous eyes. Both cpVD and cpRNFLT demonstrated significant structure function relationship in all sectors except for the nasal sector (cpVD: *P* < 0.001–*P* = 0.002, cpRNFLT: *P* < 0.001–*P* = 0.010). Correlations were strongest in the temporal and inferior temporal sectors. All mVD sectors demonstrated significant structure–function relationship with VF sensitivity with stronger association seen in the perifoveal sectors (*P* < 0.001–*P* = 0.042). GCIPL demonstrated significant relationship with corresponding VF in both the superior and inferior sectors (*P* < 0.001, *P* < 0.001). Scatter plot graphs to illustrate the sectoral structure–function relationship of cpVD and mVD with corresponding VF sensitivity are shown in Fig. 2a, b, respectively.

**Table 3:** Sectoral structure–function relationship of cpVD, cpRNFLT, mVD and GCIPL in glaucomatous eyes (n = 224).

		Estimate	*P* value*
cpVD (%)	Global	0.375 ± 0.065	** < 0.001**
	Superior	0.286 ± 0.044	** < 0.001**
	Inferior	0.577 ± 0.056	** < 0.001**
	Inferior nasal	0.339 ± 0.066	** < 0.001**
	Inferior temporal	0.527 ± 0.041	** < 0.001**
	Superior nasal	0.143 ± 0.046	**0.002**
	Superior temporal	0.372 ± 0.037	** < 0.001**
	Nasal	0.103 ± 0.063	0.106
	Temporal	0.507 ± 0.008	** < 0.001**
cpRNFLT (µm)	Global	0.077 ± 0.016	** < 0.001**
	Superior	0.062 ± 0.009	** < 0.001**
	Inferior	0.101 ± 0.012	** < 0.001**
	Inferior nasal	0.052 ± 0.015	** < 0.001**
	Inferior temporal	0.097 ± 0.009	** < 0.001**
	Superior nasal	0.026 ± 0.010	**0.010**
	Superior temporal	0.072 ± 0.007	** < 0.001**
	Nasal	0.004 ± 0.015	0.795
	Temporal	0.072 ± 0.021	**0.001**
mVD (%)	Global	0.104 ± 0.034	**0.003**
	Perifoveal superior	0.182 ± 0.037	** < 0.001**
	Perifoveal inferior	0.193 ± 0.035	** < 0.001**
	Parafoveal superior	0.097 ± 0.047	**0.039**
	Parafoveal inferior	0.098 ± 0.048	**0.042**
GCIPL (µm)	Global	0.063 ± 0.017	** < 0.001**
	Superior	0.075 ± 0.018	** < 0.001**
	Inferior	0.126 ± 0.023	** < 0.001**

cpVD: circumpapillary vessel density; cpRNFLT: circumpapillary retinal nerve fiber layer thickness; mVD: macular vessel density; GCIPL: ganglion cell-inner plexiform layer.

Significant values are shown in bold.

*Linear mixed models.

## Discussion

Previous studies have reported decreased cpVD and mVD in glaucoma patients and similar diagnostic ability between cpVD and cpRNFLT in different disease severities^10^. However, reports on the diagnostic ability of OCTA in early glaucomatous eyes were mainly conducted in small cohorts and the reported diagnostic abilities varied from moderate to high in cpVD parameters (AUROC: 0.726 to 0.965), and low to high in mVD parameters (AUROC: 0.562 to 0.92)^11–17^. Geyman et al. reported that the diagnostic ability of cpVD (AUROC: 0.907) was comparable to that of cpRNFLT (AUROC: 0.934)^17^ while Lu et al. reported that AUROC of cpVD (0.965) was higher than cpRNFLT (0.942)^12^. Chung et al. reported that AUROCs of cpRNFLT was highest (0.832), followed by GCIPL (0.805), cpVD (0.726) and mVD (0.562)^11^ in early glaucomatous eyes, which was similar to the trends reported by Rao et al.^18^ on moderate glaucomatous eyes. Our study conducted on the largest number of early glaucomatous eyes found similar trends as the reports by Chung et al.^11^.

One possible reason that the diagnostic ability of cpVD was lower than that of cpRNFLT could be that the decrease of cpVD may have occurred as a result of cpRNFLT loss and may not necessarily be a concurrent phenomenon or causal event. Lee et al. found that the area of vascular impairment highly coincided with RNFL defect position instead of following retinal vessel territories and suggested that the superficial retinal vascular decrease occurs as a secondary loss in areas with RNFL loss^21^. However, it is still controversial whether vascular damage precedes neurodegeneration in glaucomatous damage or vice versa. Another possibility is that even the newest models of OCTA may still have limitations in detecting subtle vascular changes in early glaucomatous eyes.

Correlation between sectoral cpVD and VF sensitivity in moderate glaucomatous eyes has been reported by Sakaguchi et al.^20^. They found that the correlation was strongest when the cpVD sector corresponded to the VF sector for all sectors (sr^2^ = 0.17–0.39), and the correlation was strongest in the superior temporal (ST) (sr^2^ = 0.39), temporal (T) (sr^2^ = 0.38) and inferior temporal (IT) (sr^2^ = 0.34) sector. Shin JW et al. examined the correlation of sectoral cpVD with corresponding VF sensitivity at different glaucomatous stages and found significant structure–function relationship in all sectors in moderate to severe glaucoma, whereas only the ST and IT sector showed significant relationships in early glaucomatous eyes^22^. These sectors are the most frequently affected in glaucomatous eyes^23^ and our study also showed strongest correlation in IT (Estimate = 0.527), T (Estimate = 0.507), and ST (Estimate = 0.372) sectors. In addition, our study was the first to demonstrate significant structure–function relationship of cpVD in all sectors except the nasal sector in early glaucomatous eyes.

Despite the lowest diagnostic ability of mVD, we were able to find structure function relationship with VF sensitivity in both parafoveal and perifoveal mVD in our early glaucomatous eyes. Furthermore, our results demonstrated that perifoveal mVD showed stronger structure–function relationship than parafoveal mVD. Most previous studies evaluating diagnostic ability of mVD in early glaucoma used 3 × 3 mm macular scans^11,13,14^ due to scan speed limitations, which is the same scan size as the parafoveal region of our study. Chung et al. used 3 × 3 mm macular scans and failed to find a decrease of mVD in early glaucomatous eyes and concluded that mVD may have a limited role in early glaucoma assessment^11^. However, Hou et al. reported significant decrease in parafoveal mVD in early glaucomatous eyes using 3 × 3 scans and reported similar diagnostic ability (*P* = 0.198) of mVD (AUROC: 0.73) and ganglion cell complex (GCC) thickness (AUROC: 0.78)^13^. Our parafoveal mVD results were in closer agreement with Chung et al. although significant association with visual field sensitivity was observed demonstrating that parafoveal vasculature measurements can contain useful information in early glaucomatous eyes.

With the increase in OCTA scan speed, it has become possible to obtain higher quality OCTA images of wider scan areas and we were able to use 6 × 6 mm scans for our study. Some previous studies^12,24,25^ reported higher diagnostic ability of the 6 × 6 mm macular scan in differentiating early to severe glaucomatous eyes from normal eyes compared to 3 × 3 mm scan. We also found stronger correlation with VF sensitivity in the perifoveal mVD of glaucomatous eyes, demonstrating that vascular assessment of the macula is useful even in the earlier stages of glaucoma when a wider scan area is used. Considering that damage in the macular vulnerability zone which is located in inferior macula and projects to inferior quadrant of the disc^26^ is within the 6 × 6 mm scan area, it is reasonable that our study showed a more prominent structure–function relationship in the perifoveal region. These results suggest that wider scans are likely to be more useful in evaluating early glaucomatous damage in the macular region.

Lu et al.^12^ reported comparable diagnostic ability of the perifoveal mVD to that of cpVD, cpRNFLT and GCC. However, our results showed that the diagnostic ability of the perifoveal mVD was significantly lower than other parameters (AUROC: 0.626 95% CI 0.546 to 0.705). This difference may have partially arisen because the glaucoma group in the report of Lu P et al. had worse MD (− 3.32 ± 1.42 dB) than our study and had more prominent decrease in mVD. Furthermore, the rate and temporal sequence of GCC thinning and mVD have been reported to differ at different stages of glaucoma suggesting that mVD loss is also secondary to structural thinning as it is suggested for cpVD^13,27,28^. However, further technological advances are anticipated to clarify these discrepancies in reports on early glaucomatous macular microvascular damage.

There are some limitations to our study. First, both normal tension glaucoma (NTG) and high tension glaucoma (HTG) were included in this study. Varying results have been reported on the differences of OCTA parameters between NTG and HTG^29–31^ which may have some effect on the diagnostic ability and structure function relationship reported in our study. However, the majority of the eyes included in this study were NTG eyes (approximately 70%) and the impact is not projected to be large. Secondly, highly myopic eyes were excluded from our study to match axial length with normal controls. Shin JW et al. reported that cpVD showed better correlation with VF sensitivity than cpRNFLT in moderate glaucomatous eyes of high myopia^32^. Future studies are needed to compare the impact of high myopia on diagnostic ability of OCTA. Thirdly, correlation between mVD and VF sensitivity may have been lower due to the fact that we used 24-2 VF results which only include 12 test points within the central 10 degree area^33^. Using a wider macular scan, or 10-2 VF tests may have yield higher correlation and higher diagnostic ability.

In conclusion, we found moderate diagnostic ability and significant sectoral structure function relationship of cpVD in early glaucomatous eyes. While diagnostic ability was weaker with mVD compared to cpVD, both the parafoveal and perifoveal mVD had significant association with VF sensitivity, demonstrating that subtle macular vasculature compromises are detected by OCTA at the earlier stages of glaucoma. The perifoveal region of the macular OCTA scan proved to be more useful than the parafoveal region suggesting the necessity of using wider scan areas for evaluation of early glaucomatous changes.

## Methods

### Participants

Protocols for this retrospective observational study were approved by the Research Ethics Committee of the Graduate School of Medicine and Faculty of Medicine at The University of Tokyo (Identifier: 2217). Patients gave written informed consent for their information to be stored in the hospital database and used for research at their first visit. Requirement for further written informed consent for this retrospective observational study was waived by the Research Ethics Committee of the Graduate School of Medicine and Faculty of Medicine at The University of Tokyo (Identifier: 2217). Instead, study participants were notified of the protocol posted at the outpatient clinic and were provided with the opportunity to opt-out of the study. The study protocol adhered to the tenets of the Declaration of Helsinki.

Participants of this study included consecutive normal subjects who consulted for a routine eye examination or refractive error and patients with early glaucoma or PPG (MD > − 6 dB) from the University of Tokyo Hospital (Tokyo, Japan), Yotsuya Shirato Eye Clinic (Tokyo, Japan) and Tajimi Iwase Eye Clinic (Gifu, Japan) between June 2020 and July 2021.

All participants included in this study underwent the following ocular examinations: refraction and corneal curvature radius measurements, best corrected visual activity (BCVA), axial length measurement, slit-lamp examination, intraocular pressure (IOP) measurement with Goldmann applanation tonometry, gonioscopy, fundus examination including optic nerve head examination, optic disc stereophotography, OCT and OCTA imaging. Subjects also underwent Humphrey Field Analyzer (HFA) (Carl Zeiss Meditec, Dublin, California, USA) measurements with the 24-2 Swedish Interactive Threshold Algorithm standard strategy within 3 months of the OCT and OCTA imaging.

Inclusion criteria for this study were BCVA of 20/25 or better, attainment of good quality OCT/OCTA scanning and reliable VF results (< 20% fixation loss, < 15% false negative errors and < 15% false positive errors). Patients with a history of corneal and vitreous surgery, corneal opacity, clinically significant cataract, retinal disease, and non-glaucomatous optic neuropathy were excluded from this study.

Diagnosis of each eye was conducted by at least two glaucoma specialists (KA, HS). Normal eyes had normal optic disc appearances on fundus examination and fundus stereophotographs, IOP < 21 mmHg, no abnormal VF results and no abnormal findings on slit-lamp examination and fundus examination. VF was considered to be abnormal if one of the following criteria was met. (1) the pattern deviation probability plot showed a cluster of 3 or more points with a probability of less than 5% and at least 1 point with a probability less than 1% in an expected location, (2) the pattern standard deviation had a probability of less than 5% or (3) the glaucoma hemifield test indicated that the field is out of normal limits, according to the criteria described by Anderson and Patella^34^.

Glaucomatous eyes included in this study had open angles on gonioscopy, glaucomatous change (i,e. neuroretinal rim narrowing, notching and the presence of retinal nerve fiber layer defects) of the optic nerve head on fundus examination and fundus stereophotographs, VF abnormality as previously defined^34^ with MD > − 6 dB in accordance with glaucomatous change of the optic nerve head. PPG eyes with glaucomatous optic nerve head changes but without apparent VF abnormalities on the 24-2 HFA results were also included in this study.

### OCT and OCTA imaging

OCT and OCTA imaging were performed using Cirrus HD-6000 with AngioPlex OCTA (Carl Zeiss Meditec, Dublin, CA) with a scan speed of 100,000 A-scans per second and eye tracking technology which reduces the chance of motion artifacts such as those caused by blinks and saccades. Cirrus HD-6000 is the newest OCTA model with a scan speed of approximately 1.5 times faster than previous models used in prior OCTA papers (Cirrus HD-5000 (Carl Zeiss Meditec, Dublin, CA): 68,000 A-scans per second and Angio Vue (Optovue, Inc, Fremont, California, USA): 70,000 A-scans per second). All subjects underwent 6 × 6 mm optic nerve head (ONH) cube scans, 6 × 6 mm macular cube scans, 4.5 × 4.5 mm ONH angiography scans and 6 × 6 mm macular angiography scans. Only images of optimal quality (signal strength indices > 7) without artifacts were selected. En face microvascular flow images were obtained by comparing differences in the phase and intensity information contained within sequential B-scans performed at the same area. An automatic retinal layer segmentation program was applied to each scan to obtain layer specific blood flow.

CpRNFLT, GCIPL, cpVD and mVD were measured in all subjects. CpVD was calculated as the density of the superficial capillary plexus between the inner limiting membrane (ILM) and RNFL inside a 4.5 mm diameter circle centered on the disc excluding the optic disc area. Vessel density is defined as the total area of perfused vasculature per unit area in the region of measurement and large vessels were excluded from the analysis. Superior and inferior averages provided by the commercial software and 6 Garway-Heath sectoral values^35^ calculated from the OCT raw data were used to assess sectoral structure function relationships for cpRNFLT and cpVD parameters.

MVD was calculated as the density of superficial capillary plexus between the ILM and inner plexiform layer inside a 6 mm diameter circle excluding the 1 mm diameter foveal avascular zone. The measurement area was divided into parafoveal (annulus with outer diameter 3 mm and inner diameter 1 mm) and perifoveal (annulus with outer diameter 6 mm and inner diameter 3 mm) regions which were further subdivided into four quadrants following the Early Treatment Diabetic Retinopathy Study (ETDRS) sectors. The superior and inferior quadrants of the parafoveal and perifoveal were used for sectoral analysis.

Three consecutive ONH angiography and macular angiography scans were taken on the same visit from 20 normal eyes and 20 glaucomatous eyes to assess intra-visit reproducibility.

### Statistical analysis

All data are reported as the mean ± standard deviation unless otherwise specified. ANCOVA (analysis of covariance) was used to compare parameters between normal and glaucomatous eyes adjusting for age and axial length. Categorical variables were compared using chi-square tests. Intra-visit reproducibility was evaluated by intra-class correlation coefficients. AUROCs were used to evaluate the diagnostic ability of each parameter. Comparison of AUROCs were performed using Delong tests. One eye from each subject was randomly selected for AUROC analysis and unpaired t-tests of OCT/OCTA parameters. Structure function relationships of the sectoral parameters and VF sensitivity of the corresponding regions in glaucomatous eyes were determined using linear mixed models considering inter-eye correlations. Statistical analyses were performed with commercially available software (SPSS version 27.0; SPSS, Inc., Chicago, IL, USA). Delong tests were performed with EZR (Saitama Medical Center, Jichi Medical University, Saitama, Japan), which is a graphical user interface for R (The R Foundation for Statistical Computing, Vienna, Austria). *P* values < 0.05 were considered statistically significant.

## Data Availability

The data that support the findings of this study are available from the corresponding author upon request. The data are not publicly available because they contain information that may compromise the privacy of the research participants.
